# Detection of Solitary Pulmonary Nodules Based on Brain-Computer Interface

**DOI:** 10.1155/2020/4930972

**Published:** 2020-06-15

**Authors:** Shi Qiu, Junjun Li, Mengdi Cong, Chun Wu, Yan Qin, Ting Liang

**Affiliations:** ^1^Key Laboratory of Spectral Imaging Technology CAS, Xi'an Institute of Optics and Precision Mechanics, Chinese Academy of Sciences, Xi'an 710119, China; ^2^Department of Radiology, The First Affiliated Hospital of Xi'an Jiaotong University, Xi'an 710061, China; ^3^Department of Computed Tomography and Magnetic Resonance, Children's Hospital of Hebei Province, Shijiazhuang 050031, China; ^4^BeiJing Hi-Tech Institute, Beijing 00085, China; ^5^Department of Biomedical Engineering, The Key Laboratory of Biomedical Information Engineering of The Ministry of Education, School of Life Science and Technology, Xi'an Jiaotong University, Xi'an 710061, China

## Abstract

Solitary pulmonary nodules are the main manifestation of pulmonary lesions. Doctors often make diagnosis by observing the lung CT images. In order to further study the brain response structure and construct a brain-computer interface, we propose an isolated pulmonary nodule detection model based on a brain-computer interface. First, a single channel time-frequency feature extraction model is constructed based on the analysis of EEG data. Second, a multilayer fusion model is proposed to establish the brain-computer interface by connecting the brain electrical signal with a computer. Finally, according to image presentation, a three-frame image presentation method with different window widths and window positions is proposed to effectively detect the solitary pulmonary nodules.

## 1. Introduction

Pulmonary nodules are the main pulmonary lesions. Malignant pulmonary nodules may be transformed into lung cancer, which is a serious threat to human health [1, 2]. It needs many years of clinical practice for professional doctors to make accurate diagnosis. Recently, with the development of human brain research, the research of a brain-computer interface (BCI) has become more and more popular. Vansteensel et al. [3] realize cognitive control based on BCI. Tan and Nijholt [4] establish a human-computer interaction channel. Jin et al. [5] optimize the representation of BCI stimulation targets. Myrden et al. [6] use BCI technology to realize bilateral transcranial Doppler ultrasound. The validity of P300 in an EEG signal is verified by Duvinage et al. [7]. Lin and Yang [8] establish an EEG and blinking relationship to control wheelchair movement. Do et al. [9] apply BCI technology to gait correction. Gu et al. [10] establish a semisupervised network to realize BCI online. Sun and Zhou [11] review the development of BCI and predict the development of EEG feature extraction and classification. Mikołajewska and Mikołajewski [12] establish a brain-computer interface from the perspective of children for analysis. Nijholt [13] introduces a competition and coordination mechanism to realize BCI learning. Kumar et al. [14] extract features from EEG signals and apply them to the medical field. Jeunet et al. [15] propose the standard of BCI sequence. Liu et al. [16] establish a correlation model between the P300 signal and time to realize BCI. Thomas et al. [17] establish a deep learning network to realize BCI learning. Dong et al. [18] establish an SVM algorithm to analyze multiple EEG data. Guy et al. [19] use P300 to analyze literacy signals to assist patients. Schwemmer et al. [20] use a deep neural network to simulate people's expectations of events. Abbasi [21] serves the paralyzed through BCI. Wan et al. [22] analyze the performance of BCI from the perspective of EEG. Sengupta et al. [23] establish a semisupervised neural network to realize the medical auxiliary treatment system. Wang et al. [24] propose an algorithm to enhance the useful signal strength of EEG. All the above algorithms are studied from different layers, and good results are obtained. However, there are few researches in the field of image recognition based on BCI.

Currently, the main problems and difficulties of image recognition based on BCI are as follows: (1) Based on the brain structure, it is difficult for people to stay focused for a long time, making EEG information overlapping. (2) There is a large amount of EEG data, so it is difficult to select effective features. (3) Because of the particularity of the medical image, how to build an effective response mechanism is an important issue.

In view of the above problems and difficulties, in this paper, we propose a detection algorithm of solitary pulmonary nodules based on a brain-computer interface. (1) The time-frequency feature fusion model is constructed to enhance the signal identification. (2) A multifeature network based on deep learning is proposed. (3) Through the training of doctors and ordinary people, an effective response mechanism is proposed.

## 2. Algorithm

**Figure 1:**
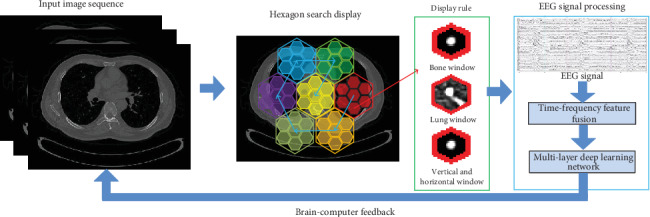
The algorithm flow chart.

To solve the above problems, we design the algorithm flow as shown in Figure 1. First, the image is blocked and displayed according to specific rules. Then the time-frequency feature fusion model is constructed to enhance the signal identification. An effective feature extraction method based on the stacking fusion model is proposed. A complete brain-computer interface is constructed to detect solitary pulmonary nodules.

### 2.1. Time-Frequency Feature Fusion

EEG signals have multiple frequencies, from which useful information needs to be extracted. The MEMD (multivariable electromagnetic mode decision) algorithm can ensure that signals from different sources get decomposition results matching each other in terms of quantity and frequency. It has advantages in processing multichannel EEG signals [25].

MEMD can expand the single variable EMD in many dimensions, realize the joint analysis of multiple vibration components of high-dimensional signals, and avoid the modal aliasing problem of standard EMD. The specific algorithm is described as follows.

*Data sampling*: calculate the set {*P*^*x*^(*t*)}_*k*=1_^*K*^ of mappings of the input signal **V** along the direction vector **x**. Find the time point *t*^*x*^ corresponding to the maximum value in {*P*^*x*^(*t*)}_*k*=1_^*K*^. Obtain the multivariate envelope curve {*e*^*x*^(*t*)}_*k*=1_^*K*^.

Calculate the mean value of envelope curve: *m*(*t*). Calculate *d*(*t*) = *x*(*t*) − *m*(*t*) until stop if *d*(*t*) meets IMF criteria. Otherwise, it will iterate.

Hilbert transform [26] is applied to each channel of the obtained brain-computer signal to decompose the signal:

(1) $$\begin{array}{c} Y_{k}\left(t\right)=\frac{1}{\pi }\kappa \int_{-\infty }^{\infty }\frac{u_{k}\left(t^{\prime }\right)}{t-t^{\prime }}dt, \end{array}$$

(2) $$\begin{array}{c} Z_{k}\left(t\right)=u_{k}\left(t\right)+{jY}_{k}\left(t\right), \end{array}$$

where *κ* is the Cauchy value and *u*(*t*) is the input signal. Rewrite Equation (2) as follows:

(3) $$\begin{array}{c} Z_{k}\left(t\right)=a_{k}\left(t\right)e^{j\theta_{k}\left(t\right)}, \end{array}$$

where *a*_*k*_(*t*) is the amplitude and *θ*_*k*_(*t*) is the phase.

(4) $$\begin{array}{c} \left\{\begin{array}{c} \theta_{k}\left(t\right)=\arctan\left(\frac{Y_{k}\left(t\right)}{u_{k}\left(t\right)}\right),\\ w_{k}\left(t\right)=\frac{\text{d}\theta_{k}\left(t\right)}{\text{d}t}. \end{array}\right. \end{array}$$

Each component of the EEG signal is expressed as

(5) xt=Re∑k=1Kaktejθkt.

The Hilbert spectrum is defined as

(6) Hw,t=Re∑k=1Kaktej∫wktdt.

Solve the Hilbert energy spectrum (IES) and the Hilbert marginal spectrum (MS).

(7) $$\begin{array}{c} \left\{\begin{array}{c} \text{IES}\left(t\right)=\int_{w_{1}}^{w_{2}}H^{2}\left(w,t\right)\text{d}w,\\ \text{MS}\left(w\right)=\int_{0}^{T}H\left(w,t\right)\text{d}w, \end{array}\right. \end{array}$$

where *w*_1_ and *w*_2_ correspond to different frequency bands of EEG.

Calculate the mobility parameter Mob in the Hjorth parameter.

(8) $$\begin{array}{c} \text{Mob}=\sqrt{\frac{\text{Var}\left(y^{\prime }\left(t\right)\right)}{\text{Var}\left(y\left(t\right)\right)}}. \end{array}$$

The mean value and standard deviation are calculated as the feature **F**_*t*_ in the time domain, and the feature **F**_*s*_ in the frequency domain is calculated in the same way.

Sample entropy is introduced to characterize the nonlinear dynamic coupling characteristics of EEG signals.

The input sequences *u*(*i*) and *v*(*i*) are reconstructed into vectors:

(9) $$\begin{array}{c} \begin{array}{c} \left\{\begin{array}{c} X\left(i\right)=\left[u\left(i\right),\cdots ,u\left(i+m-1\right)\right],\\ Y\left(i\right)=\left[v\left(i\right),\cdots ,v\left(i+m-1\right)\right], \end{array}\right.\\ \;i\in \left[1,N-m+1\right]. \end{array} \end{array}$$

Calculate the distance

(10) $$\begin{array}{c} d\left(i,j\right)=\left|u\left(i+k\right)-v\left(j+k\right)\right|\;k=0\cdots m-1, \end{array}$$

and statistical proportional mean

(11) $$\begin{array}{c} B^{m}\left(r\right)=\frac{1}{N-m}\overset{N-m}{\underset{i=1}{\sum }}B_{i}^{m}\left(r\right),\\ B_{i}^{m}\left(r\right)=\frac{N\left(d\leq R\right)}{N\left(d\right)}, \end{array}$$

where *N*(·) is the number of qualified pixels and *R* is the conditional threshold. The cross sample entropy can be defined as

(12) $$\begin{array}{c} C\left(m,r\right)=-\ln\left(\frac{B^{m+1}\left(r\right)}{B^{m}\left(r\right)}\right). \end{array}$$

Characterize the relative complexity of two sequences.

To characterize the phase coupling relationship, we calculate

(13) $$\begin{array}{c} Q=\frac{1}{N}\left|\overset{N}{\underset{i}{\sum }}\exp\left(j\left(\theta_{1}-\theta_{2}\right)\right)\right|. \end{array}$$

Similarly, calculate the frequency locking value:

(14) $$\begin{array}{c} S=\frac{1}{N}\left|\overset{N}{\underset{i}{\sum }}\exp\left(j\left(w_{1}-w_{2}\right)\Delta t\right)\right|, \end{array}$$

where *N* is the sampling frequency, *θ*_1_ and *θ*_2_ are the phase information, and *w*_1_ and *w*_2_ are the frequency information. The single channel time-frequency characteristics of the EEG signal are as follows:

(15) $$\begin{array}{c} F=\left\{F_{t},\cdot F_{t},\cdot Q,\cdot S\right\}. \end{array}$$

*F* is used as an EEG signal feature for the following calculation.

### 2.2. Multilayer Deep Learning Network

An EEG signal has many signals, so it *is* difficult to extract useful information by a single learning framework. A stacking algorithm [27] can realize the efficient utilization of training data. The main idea of the algorithm is to train several different basic learners based on the initial training data set and then generate a new data set to train the next layer of learners according to the output of the primary learner as the input. The tag value of each training data is unchanged. In practice, in order to prevent overfitting, we often use cross validation or leave one method to generate the training samples of the next level of the learner with the samples not used in the training of the basic learner, and the basic learner uses different learning algorithms for generation.

In order to improve the recognition ability and generalization of the model, we use five simple heterogeneous learning devices, including SVM, random forest [28], logistic regression [29], KNN [30], and AdaBoost [31] to build a two-tier stacking integrated learning model.

The proposed algorithm in this paper adopts the deep learning framework. The training set and test set are very important. The ratio of the training set and the test set is 1 : 1.

Considering the phenomenon of overfitting, the training set of the secondary learners is obtained by using the method of half-fold cross validation, that is, each primary learner is used to train the current compromised training set in each compromise, and then the output of the current compromised verification set is predicted. When the cross validation is completed, the mapping of all the data in the original training set is completed and the secondary training set is generated.

**Figure 2:**
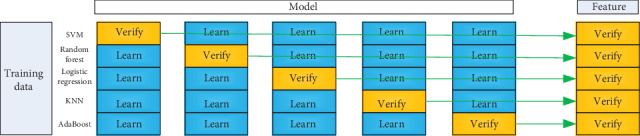
Deep learning network based on multiple features.

Take the average value after each predicted test set. The execution process of the same primary learner cross validation is shown in Figure 2. First, the original training set is divided into five parts. In each iteration, four parts of the data are taken as the training set in turn, and the primary learner is trained. Then, the remaining training data and all test data sets are predicted, and the prediction results are saved. In this way, after five iterations, each primary learner is trained five times, and each data divided initially is predicted. All test data is predicted. A new training data matrix with the number of rows equal to the number of training set samples and the number of columns equal to the number of primary learners is obtained, which is the input to the secondary learner as training data. After the training of the secondary learner, the new test set is predicted and the final output is obtained.

## 3. Data Acquisition and Processing

The data studied in this paper can be divided into brain data and pulmonary image data. It includes data acquisition, annotation, and processing.

### 3.1. Data Acquisition and Presentation

The image sequence is from the International Early Lung Cancer Action Project database [32, 33], and the lung CT images were collected by the hospital. Image presentation is on a 21-inch LCD. The subjects sit in a comfortable posture, and the distance between their eyes and the center of the screen is about 80 cm. EEG signal acquisition uses a 64-channel brain product EEG. According to the international standard 10-20 system, electrode placement ensures effective coverage of all interested cortical areas. The sampling frequency of the EEG signal is 1000 Hz. Before the experiment, make sure that the impedance of each electrode is below 5 k*Ω*. In order to reduce the possible EMG signal crosstalk, the subjects are required to reduce head activity as much as possible after completing the EEG electrode arrangement.

### 3.2. Data Annotation

The image sequence of a solitary pulmonary nodule is labeled by two professional doctors using the independent blind marking method. The gold standard of pulmonary nodules is selected.

All the data are marked by two professional doctors in accordance with the blind mark method, and disputed annotations are arbitrated by a third expert.10 groups of lung CT images are collected in the experiment, which include 3200 frames in total. EEG signals include 100 sets.

### 3.3. Data Processing

In order to increase the locality of the image sequence, maximize the awareness of spatial context, and minimize the vertigo of the interpreter, the hexagon search path algorithm is adopted [34]. The hexagon search path algorithm decomposes a two-dimensional hexagon mesh into a nested set of approximate self-similar patterns with approximate hexagon symmetry. From the largest hexagon in the image to the smallest hexagon, it is similar to a Piano De Karl space to fill the whole image space. Through the hexagon search path algorithm.

The EEG signal is preprocessed as follows: (1) Resampling: reduce the EEG signal frequency to 256 Hz in order to improve processing speed and remove interference. (2) Select the common average reference surface: the average value of all electrodes is used as the common average reference in this experiment. (3) Filtering: this is done because the power frequency signal and high-frequency interference will be mixed in the EEG acquisition process, and the frequency of the EEG signal with research significance based on the oddball experimental paradigm is generally less than 50 Hz. Thus, the passband frequency of a 0.5~48 Hz filter is adopted in this experiment.

## 4. Experiment and Result Analysis

**Figure 3:**
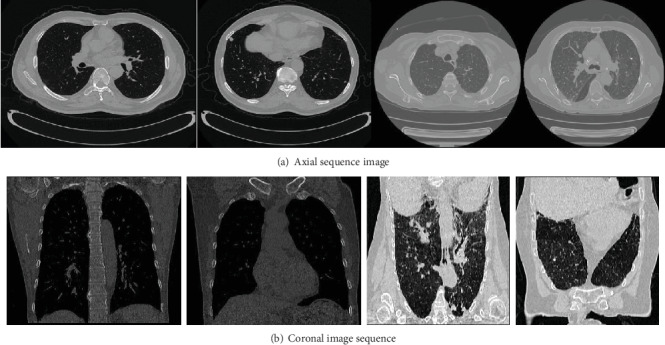
Experiment data presentation.

The subjects are divided into two groups, six in each group (three males and three females, average age 35 years). Group 1 consists of professional radiologists. Group 2 consists of people who had not participated in video training. There are 20 groups of image data, among which the coronal image sequence is generated by coronal sequence, as shown in Figure 3.

Each experimenter needs to complete two sessions, with a rest time of no more than five minutes between each session. Each session contains several blocks. Before the start of the block, the flashing red cross symbol is displayed in the center of the screen to remind the subjects to pay attention, and the two types of stimulus images appear in a proportional order.

### 4.1. Saliency and Specificity Verification

The significance of response and the specificity of brain response are the premise of brain-computer interface research. Because of the particularity of the medical image, the traditional brain-computer interface criterion cannot be directly applied to lung CT image detection. Therefore, we study the significance of test response and the specificity of brain response.

#### 4.1.1. Test Response Saliency

According to the CT image data of pulmonary nodules, in order to verify whether there is significant difference in behavior of the subjects, we have made statistics on the recognition effect of group 1 and group 2.

Sensitivity (SEN), specificity (SPE), and false positive fraction (FPF) are introduced to measure the performance of different algorithms [34]:

(16) $$\begin{array}{c} \text{SEN}=\frac{\text{TP}}{\text{TP}+\text{FN}},\\ \text{SPE}=\frac{\text{TN}}{\text{TN}+\text{FP}},\\ \text{FPF}=\frac{\text{FP}+\text{FN}}{\text{TP}+\text{FP}+\text{TN}+\text{FN}}, \end{array}$$

where TP is True Positive, FN is False Negative, FP is False Positive, and TN is True Negative.

In this paper, the parameter setting of the comparison algorithms is based on the parameter range mentioned in the corresponding article to find the optimal parameters.

From Table 1, the detection effect by specially trained doctors on pulmonary nodules is much higher than that by group 2. Furthermore, it is confirmed that there is significant difference in behavior among the pulmonary nodule test subjects.

**Figure 4:**
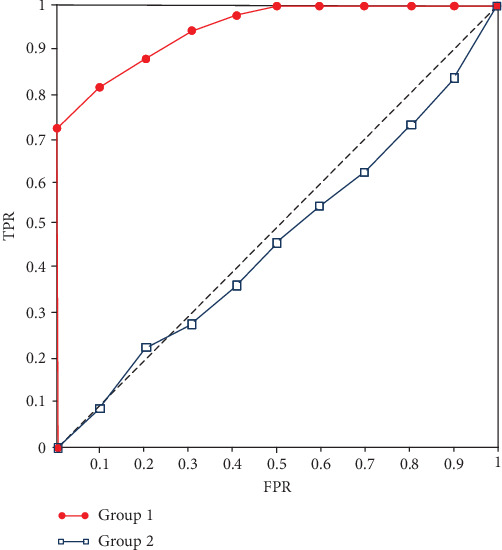
Recognition ROC figure.

To visually demonstrate the detection effects of different groups, an ROC curve is shown in Figure 4. We can see that the recognition effect of group 1 is much higher than that of group 2.

#### 4.1.2. Brain Response Specificity

**Figure 5:**
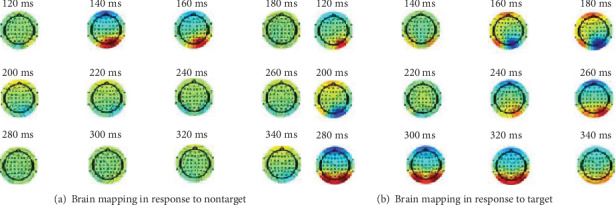
Brain response mapping.

The EEG data of subjects under relevant tasks are collected, and the specific brain response information of subjects to target and nontarget images is studied through the active participation of subjects in the target detection task. The EEG data of each subject are analyzed independently. Compared with the nontarget image, the brain activity of the prefrontal cortex area is more intense when the target image is observed, which is in line with P300 characteristics. That is to say, the EEG amplitude of the prefrontal cortex area would be larger than that of the normal stimulus sequence about 300 ms after the abnormal stimulus sequence is observed. The difference of the EEG amplitude is convenient to distinguish two kinds of data signals to judge the corresponding CT images of pulmonary nodules. In Figure 5, it is the brain topographic map of the target and nontarget images observed by one of the subjects in the experimental group at different time points. It can be concluded that the amplitude of the prefrontal cortex in the observation target image EEG data between 280 and 320 ms is larger than that in other time periods, while the EEG data changes little in the observation of nontarget images.

### 4.2. Image Presentation

The CT image of the lung has the characteristics of medicine and anatomy, which leads to a great difference between CT images and traditional images. In order to obtain EEG signals better, we start experiments from the time of image display, the proportion of target and background display, the way of image presentation, and the times of image repetition.

#### 4.2.1. Display Time

The display time of an image will directly affect the corresponding effect on the brain. Fast sequence visual presentation is an experimental model for the detection of attention time characteristics [35]. In the fast sequence visual presentation paradigm, a series of target stimuli are placed in the background stimuli, which are fixed in the same specific position of the screen and presented in a specific period of time. Because of the particularity of the medical image, we need to further verify the effect of the display time. For this reason, we use five representative image sequences to select different display times to verify the effect.

**Figure 6:**
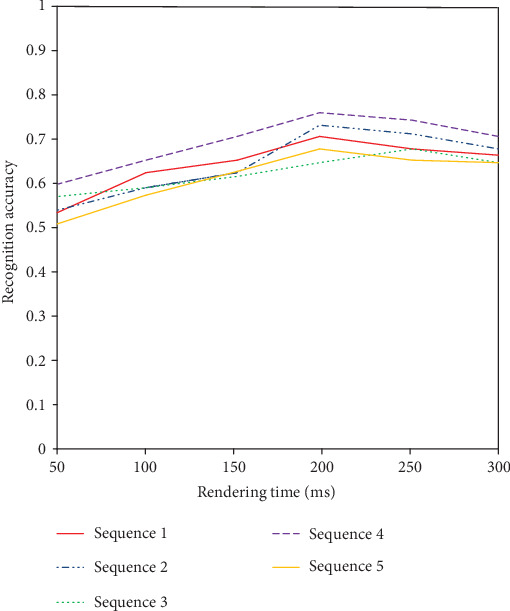
Time-recognition accuracy graph.

The recognition accuracy of 50~250 ms is shown in Figure 6. With the increase of the presentation time, the recognition rate shows an upward trend within 50-200 ms. However, with the increase of presentation time, when it is more than 200 ms, the recognition rate will decrease due to the weakening of the brain signal. So far, in this paper, we select the optimal 200 ms as the presentation time.

#### 4.2.2. Target to Background Image Number Ratio

**Figure 7:**
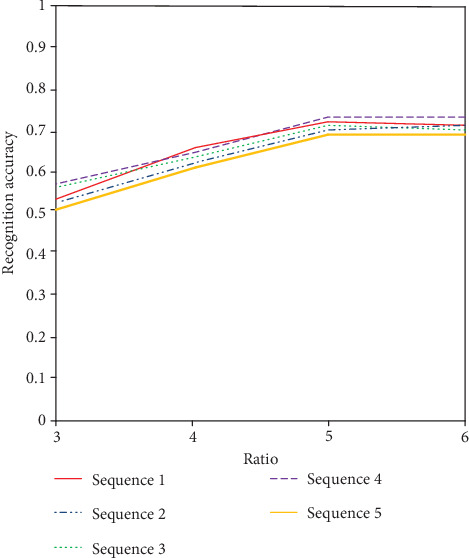
Ratio-recognition accuracy graph.

According to the oddball paradigm [36], random presentation of two stimuli acting on the same sensory channel should ensure that there is a great difference in the probability of interclass stimulus presentation under the relevant potential. Therefore, we adjust the ratio of the number of pulmonary nodule images to the number of pulmonary nodule images under the condition of a 200 ms display. As shown in Figure 7, when the ratio of the target to the background increases, the signal stimulation produced by the brain tends to increase. When the ratio of the target to background reaches 1 : 5, it tends to be stable. So far, in this paper, we select the optimal ratio of 1 : 5 as the number of target and background images.

#### 4.2.3. Number of Repetitions

Visual expert object recognition is a complex process of perception and cognition, including the dynamic interaction of low-level perception and high-level cognitive components, which involves the close coupling of perception, memory, attention, and semantics. At the central level, multiple subsystems interact and work together to support visual experts to complete object recognition tasks. For medical image interpreters, each of them has accumulated rich experience through long-term and high-intensity purposeful training, and their behavior characteristics are significant. The accuracy of EEG classification can be improved by presenting the same image multiple times. For this reason, we compared the effect of different repetition times on the recognition accuracy.

**Figure 8:**
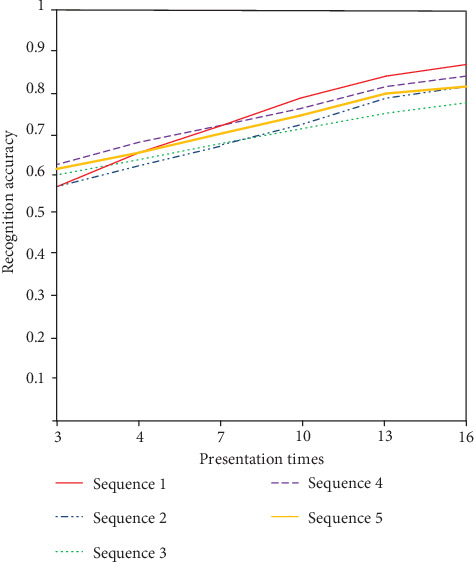
Presentation time-recognition accuracy relationship graph.

As shown in Figure 8, with the increasing number of image repetitions, the accuracy of EEG interpretation increases correspondingly, and the curve rises and slows down when the image is repeated 13 times. It can be seen that for the same subject, repeated image presentation can improve the signal-to-noise ratio of EEG data and provide more effective brain response information.

#### 4.2.4. Display Mode

Lung CT image data is 16 bits; an ordinary display cannot display all its information on a single frame image. To verify different image display modes, we define the following modes:

- *Mode 1*: the display of window width and window position is set based on the average value of a single frame CT image.
- *Mode 2*: window width and window position display mapped by the lung window.
- *Mode 3*: based on the lung window mapping, two images are generated by floating up and down, that is, three images are displayed.

The bone window, lung window, and vertical and horizontal window have a display mode with a fixed mapping range. No human-computer interaction is required. When the image is inputted, it will be automatically displayed according to the mapping rules.

**Figure 9:**
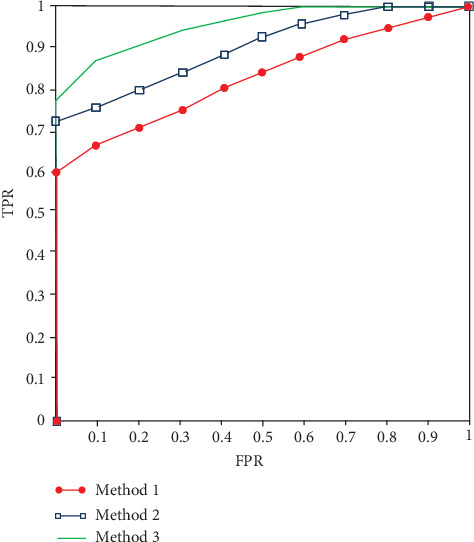
ROC curves of different methods.

As shown in Figure 9, due to the limited information obtained in Mode 1, the features of pulmonary nodules are not obvious, which lead to recognition failure. For Method 2, under the premise that the best window of the lung can be observed, the shape of the lung can be displayed better, but the recognition of pulmonary nodules with an unclear boundary still fails. Inspired by the above two methods, Mode 3 displays the information image of multiple windows and wide windows, and the tested person can make a comprehensive judgment, which has a good effect.

### 4.3. Classification Effect Comparison

**Figure 10:**
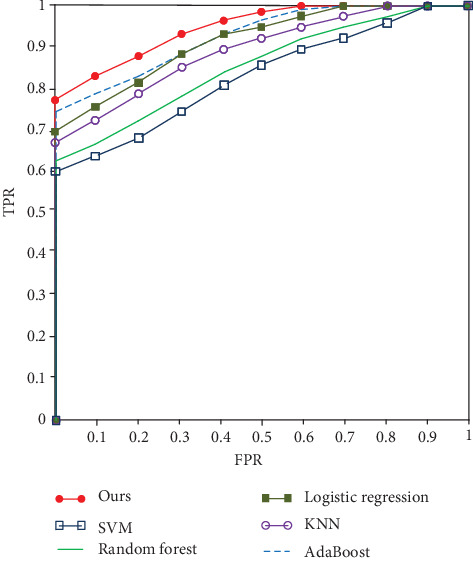
ROC curves of different algorithms.

To verify the proposed algorithm classification effect, we compare different algorithms, as shown in Figure 10. SVM [18] establishes a multilevel structure to decompose the input signal by wavelet, which has a certain effect on EEG signal classification. Random forest [28] integrates spatial information and polarizes information to realize signal classification. Logistic regression [29] extends the algorithm of polynomial logistic regression to the semisupervised learning of posterior class distribution to improve the classification performance. KNN [30] introduces convolution to the EEG image for classification. AdaBoost [31] combines fuzzy entropy, sample entropy, approximate entropy, and spectral entropy to realize EEG signal classification.

## 5. Conclusion

We studied the process of detecting solitary pulmonary nodules by physicians from the aspects of pulmonary nodule imaging and physicians' EEG response. A time-domain and frequency-domain model is proposed to extract EEG features. A multilayer feature fusion model is proposed to verify the feasibility of the brain-computer interface in detecting pulmonary nodules.

## Figures and Tables

**Table 1:** Recognition effect of each group.

Group	SEN	SPE	FPF	AUC
1	56%	92%	16%	0.89
2	17%	80%	32%	0.43

## Data Availability

All used data is within the paper.
